# Behavioral Interventions as an Adjunctive Treatment for Canine Epilepsy: A Missing Part of the Epilepsy Management Toolkit?

**DOI:** 10.3389/fvets.2019.00003

**Published:** 2019-01-28

**Authors:** Rowena M. A. Packer, Sarah L. Hobbs, Emily J. Blackwell

**Affiliations:** ^1^Royal Veterinary College, London, United Kingdom; ^2^Bristol Veterinary School, University of Bristol, Bristol, United Kingdom

**Keywords:** canine, epilepsy, behavior therapy, relaxation, drug-resistant

## Abstract

Epilepsy is a common, complex and often challenging neurological disorder to treat in the dog, with 20–30% of dogs resistant to conventional medical therapies, and associated with cognitive and behavioral comorbidities and early death. Behavioral interventions are an emerging area of focus in the adjunctive treatment of drug-resistant human epilepsy patients, with studies indicating positive effects of a variety of interventions including relaxation-based techniques and behavioral therapy interventions. Behavioral interventions have the potential not only to improve seizure control, but also improve behavioral comorbidities and general quality of life in this hard to treat patient group. Despite striking similarities between human and canine epilepsy patients, including the recognition of co-morbid anxiety in epilepsy patients, behavioral interventions have yet to be studied in dogs. This is compounded by several licensed psychopharmaceutical agents for dogs being contra-indicated in epilepsy patients. We present evidence from human studies of the efficacy of behavioral interventions to improve seizure control, psychiatric comorbidities and quality of life, and propose that adapting such interventions for canine patients may be a valuable addition to the epilepsy management toolkit. There is a need for multi-center, double-blinded, placebo-controlled trials to confirm the effects of behavioral interventions on seizure frequency in veterinary medicine. In the absence of such evidence to date, the use of established behavioral medicine techniques to reduce stress and improve the mental health of these often sensitive and challenging patients is advocated, with a greater role for behaviorists in the management of epilepsy patients alongside neurologists and general practitioners.

## Canine Epilepsy: Current Therapies and Challenges

Epilepsy is the most common chronic neurological presentation in first opinion practice for domestic dogs, with previous epidemiological studies estimating a prevalence of 0.6% of dogs affected in the UK alone (1). Epilepsy is a major risk to the health and welfare in dogs, with dogs at risk of developing behavioral changes such as anxiety and attention-deficit hyperactivity disorder (ADHD)-like behavior, cognitive impairments, reduced quality of life (QoL) from anti-epileptic drug (AED) side effects, complications of AED treatments and early death (2–7). Between 20 and 60% of dogs with IE die as a direct consequence of epilepsy (8). The condition is early onset, with most dogs experiencing their first seizure between 1 and 4 years of age, and is lifelong (9), requiring chronic medication. Medication with AEDs poses a fine balance between benefits and harms, with potentially adverse welfare consequences due to unpleasant side effects including excessive hunger, thirst, restlessness, lethargy, and ataxia (4). Their impact upon QoL may be considerable, with AED side-effects being cited as an important consideration for owners of epileptic dogs (4).

Despite advances in pharmacotherapy for canine idiopathic epilepsy (IE), with an increasing number of AEDs available to veterinarians managing these often challenging patients, a large proportion of this patient population continue to experience seizures while taking AEDs. At present, only a minority of canine epilepsy patients achieve significant periods of seizure freedom (10). Overall response rates (defined as >50% reduction in seizure frequency) to the first, second and third AEDs have been reported as 37.2, 10.7, and 6.1%, respectively (11); and thus many dogs continue to seizure chronically despite polytherapy. This parallels findings in human neurology, where up to 30% of epilepsy patients continue to seizure despite taking AEDs (12). Although AEDs remain the mainstay of epilepsy treatment in dogs, seizure control is often incomplete and their use is associated with a variety of quality of life inhibiting side effects including sedation, ataxia, polyphagia (5). Indeed, a recent meta-analysis of 90 canine epilepsy studies identified adverse effects associated with all nine of the most commonly used AEDs (13). The discovery of adjunctive therapies that effectively reduce seizure frequency in canine epilepsy patients, while avoiding additional side effects is of high priority. Non-pharmacological seizure control strategies are becoming increasingly common in both canine and human epilepsy, including dietary interventions [e.g., the ketogenic diet; (14, 15) and the use of dietary supplements (16)], and implantable neuromodulation devices [e.g., vagal nerve stimulators; (17, 18)]. Although non-pharmacological therapies including dietary and neuromodulation interventions have been demonstrated to improve seizure frequency in some patients, others still fail to achieve a significant level of seizure control following these adjunctive therapies (commonly considered a >50% reduction in dogs), and thus the search for additional adjunctive therapies for the epilepsy management tool-kit continues.

An emerging area in adjunctive therapies for drug-resistant human epilepsy patients is behavioral and psychological therapies. These interventions are based on accumulating evidence from animal models and clinical studies that there is a relationship between behavioral, physiological and psychological states and the probability of seizure occurrence, with a bidirectional relationship between seizures and psychological states (19). Interventions that alter behavioral or psychological states have been tested to determine whether they can reduce the occurrence of seizures in people with epilepsy, with success found with a variety of interventions. To date, this type of intervention for drug-resistant canine epilepsy patients remains entirely unexplored to the authors' knowledge, but offer much promise. There are striking similarities between canine and human epilepsy patients, with shared characteristics including spontaneous drug-resistance (20) and behavioral and cognitive comorbidities (2, 6, 7, 21). Many of the same drug and non-drug therapies (e.g., the MCT diet) have proven efficacious in both species, and as such, exploring behavioral interventions in dogs appears both logical and overdue. Potential benefits of behavioral interventions include their relatively low cost, lack of side effects and contra-indications with existing medications, and their non-invasive nature (22). As recently highlighted by Kanner (23), the treatment of epileptic seizure disorders is not restricted to the achievement of seizure-freedom, but must also include the management of co-morbid psychiatric and cognitive comorbidities (23). With the increasing recognition of behavioral and cognitive comorbidities in dogs with epilepsy (2, 6, 7, 21), the potential for behavioral interventions improving both seizure control and behavioral clinical outcomes warrants investigation in this area in canine patients.

## Evidence for Behavioral Interventions for Epilepsy

Behavioral treatments for epilepsy in humans have been discussed since 1977 (24), but have not been subjected to investigation in rigorous clinical trials until recently, with the development of psychosocial treatment programs not keeping pace with the development of medical treatments (25). Despite this, behavioral techniques to control seizures have a long history with a multitude of empirical studies published on a variety of interventions. Many of these behavioral interventions target stress. Stress is increasingly recognized to have a major role in epilepsy, including: (i) acting as a risk factor for the development of epilepsy, (ii) being a trigger for the occurrence of seizures in some individuals with epilepsy, and (iii) exacerbating seizure frequency, and/or being an important component of a prodrome preceding a seizure in some individuals with epilepsy (26). Recent research has demonstrated that epilepsy is associated with elevated stress in both dogs with epilepsy and their owners (27); however, measures to counteract stress have yet to be explored in this population. Behavioral interventions to reduce stress and improve emotional state for people with epilepsy have included relaxation exercises including yoga and progressive muscle relaxation, biofeedback and cognitive behavioral therapy, which will now be briefly reviewed. Evidence is additionally summarized in Table 1.

**Table 1:** Evidence of the effects of behavioral interventions on seizure control in human epilepsy patients.

**Type of intervention**	**References**	**Sample**	**Study design**	**Intervention groups**	**Baseline seizure frequency**	**Seizure frequency post-intervention**	**Other outcome measures**
Progressive muscle relaxation	Haut et al. (26)	Medication-resistant focal epilepsy 18+ years of age Exclusion of patients with history of status epilepticus	Multicenter, double-blinded randomized controlled trials	Progressive muscle relaxation (PMR) + diaphragmatic breathing (*n* = 32) Focused-attention activity with extremity movements (*n* = 32)	Seizures per month: PMR = 11.25 (9.6) Focused attention = 11.59 (20.42)	Seizure frequency reduced: PMR = −29% Focused attention = −25% Seizure reduction by month data available	PMR associated with self-reported stress reduction
	Rousseau et al. (28)	14+ years of age. Minimum of six seizures during 3 week baseline period	Two phase experimental study	Relaxation therapy (Group 1, *n* = 4) Sham treatment (sitting quietly 20 min twice a day, group 2, *n* = 4). Group 2 subsequently additionally had relaxation therapy (*n* = 4)	Baselines described for each participant individually	% reduction described for each participant individually	Improved well-being e.g., improved sleep, feeling less tense and/or aggravated during the day. Feeling more in control of epilepsy and less afraid of seizures.
	Dahl et al. (29)	Adults with drug-resistant epilepsy Minimum of one seizure per month	Randomized two phase experimental study	Experimental Phase Group 1, contingent relaxation (CR) Group 2, supportive therapy (ATG, attention-placebo) Group 3, no treatment (NT) Non-experimental Phase Groups 2 & 3 received CR.	Baseline averages described for each participant individually	Experimental phase (10 week follow-up): Group 1 = −66% reduction Group 2 = +68% increase Group 3 = −2% reduction	Participants felt greater control over their epilepsy and ability to predict seizures made them less dangerous.
	Puskarich et al. (30)	Minimum of six seizures during 8 week baseline	Randomized controlled trial	Progressive relaxation training (PRT, *n* = 13) Quiet sitting (QS, *n* = 11)	QS average = 10.3 PRT average = 17.0	QS = 10.0 (−3% reduction) PRT = 12.1 (−29% reduction)	No other measures reported
	Whitman et al. (31)	Adults with minimum of six seizures during 8 week baseline	Repeated measures	PRT twice a day 8 weeks (*n* = 12)	Mean seizure frequency = 20.3	Mean seizure frequency: Two-month follow-up = 14.8 Four-month follow-up = 13.9 Six-month follow-up = 12.5	No participant-related measures reported
Yoga	Sathyaprabha et al. (32)	Chronic drug-resistant epilepsy and healthy controls	Randomized controlled trial with repeated measures	Yoga = (*n* = 18) Exercise = (*n* = 16) Healthy controls = (*n* = 142)	Yoga group = 7.2 ± 1.31 Exercise group = 6.4 ± 0.51	Yoga group = 5.7 ± 0.91 Exercise group = 7.1 ± 1.4	Automatic dysfunction decrease in yoga group
	Lundgren et al. (22)	Adults 18–55 years of age with drug-resistant seizures. Minimum of three seizures in 3 months	Randomized controlled trial with repeated measures	Acceptance and commitment therapy (ACT) = (*n* = 10) Yoga = (*n* = 8)	Frequency data described for each individual participant	Frequency data described for each individual participant	Increased QoL in both groups
Biofeedback	Nagai et al. (33)	Drug-resistant epilepsy. 16–60 years of age Average minimum two/three seizures per month over 3 months	Two group, single blind, randomized controlled trial	Galvanic Skin Response (GSR) = (*n* = 10) Sham control = (*n* = 8)	Frequency data described for each individual participant	GSR mean % change in seizure frequency = −49.26% decrease Sham mean % change = +24.59% increase	No other measures reported
Behavioral/cognitive therapy	Gillham (34)	Fifty nine adults with poorly controlled epilepsy Average of minimum two seizures over 2 months	Balanced cross-over design	Group 1 (*n* = 19) no significant “psychological disorders”: “self-control of seizures” (Treatment A) Group 2 (*n* = 21) had significant “psychological disorders”: Treatment A followed by “alleviation of psychological disorder” (Treatment B) Group 3 (*n* = 19) had significant “psychological disorders”: Treatment B followed by Treatment A		No significant differences in seizure frequency between the three groups Mean improvement baseline to week 42 for all 59 patients = −32.95%	Groups 2 and 3 showed significant improvements in self-reporting of depression and anxiety after treatment.
	Spector et al. (35)	Adults with intractable seizures	Uncontrolled AB group design, repeated questionnaires	Group-based (*n* = 7)	Mean seizures per week = 5.0 (range 3–8)	Average −74% reduction	No change in psychological or psychosocial measures
	McLaughlin and McFarland (36)	Adults aged over 60 years of age	Randomized controlled trial, with repeated measures (questionnaires)	CBT group (*n* = 18) Control group (*n* = 19)	Program group = 6.33(6.62) per month Control group = 4.95 (7.43) per month	Program group: post-treatment = 3.68 (3.73); Three month follow-up = 1.39 (1.82) Control group: post-treatment = 5.00 (6.98); Three month follow-up: 4.42 (6.56)	Both groups reported lower levels of depression and improved psychosocial functioning
	Au et al. (37)	Adult patients. Exclusion criteria: active serious medical disorders, psychotic features, severe mental deficiency, neurosurgery in last 12 months	Matched groups	Treatment group (*n* = 8) “Waitlist” control group (*n* = 9)	Treatment = 3.71 (1.82) Control = 3.48 (2.23)	Treatment = 3.21 (1.46) Control = 3.78 (2.51)	Treatment group reported improved QOL and self-efficacy
	Tan and Bruni (38)	Adult outpatients with uncontrolled epilepsy and significant “psychosocial problems” (e.g., anxiety and/or depression)	Randomized controlled trial	Weekly group therapy: Cognitive Behavior Therapy group (CBT, *n* = 8) Supportive Counseling (SC), attention-placebo control group, *n* = 10) Waiting List Control (WL, *n* = 9)	Scores from 2–4 week baseline period. CBT: 10.75 SC: 1.53 WL: 4.64	Post-treatment: CBT: 15.60 SC: 1.63 WL: 6.22 Follow-up: CBT: 9.06 SC: 2.01 WL: 6.00	Improvements in “psychological adjustment” in the Cognitive-Behavior Therapy and Supportive Counseling groups.

*ACT, Acceptance and Commitment Therapy; ATG, Attention-placebo group; CBT, Cognitive Behavior Therapy; CR, Contingent Relaxation; GSR, Galvanic Skin Response; NT, No treatment; PMR, Progressive Muscle Relaxation; PRT, Progressive Relaxation Training; QOL, Quality of Life; QS, Quiet Sitting; SC, Supportive Counseling; WL, Waiting List Control*.

### Relaxation-Based Techniques

Stress management techniques have been demonstrated to show strong preliminary evidence in seizure reduction in people with epilepsy. A well-studied technique is progressive muscle relaxation (PMR) which involves tensing and releasing different muscle groups in turn. In a recent multicenter randomized controlled trial (RCT), *n* = 66 adults with refectory epilepsy were randomized into one of two interventions; PMR with diaphragmatic breathing or control focused-attention activity with extremity movements. Participants underwent 12 weeks of double-blind treatment, and seizure frequency during this period was compared to the 8 week baseline phase before treatment commenced. In both groups, seizure frequency was significantly reduced from baseline; however, there was no difference in the efficacy of the two groups (26). PMR was more effective in reducing self-reported stress than focused attention (26). Prior to this RCT, several smaller studies demonstrated the promise of this technique. In a small study of epilepsy patients (*n* = 8), half underwent 3 weeks of PMR while half in the control arm underwent 3 weeks of quiet sitting, followed by 3 weeks of PMR. All subjects showed decreases in seizure frequency from baseline to treatment and improvements in well-being (28). In a further study of *n* = 18 adults with drug-resistant epilepsy, patients were split into three groups: contingent relaxation, attention control, and no-treatment (control) for 6 weeks. Patients in the contingent relaxation group were instructed to apply PMR techniques to situations or feelings associated with high risk of seizure activity. Results indicated that seizure frequency was significantly reduced in the PMR group (29). A further study compared the 8 week baseline seizure frequency for 12 participants with three subsequent 8 week follow-up periods, post PMR training. During the intervention period, PMR was practiced twice a day. By the third follow-up time point, the median seizure frequency for the group had decreased by 54% (31). Finally, in a study of *n* = 24 adults with epilepsy, a seizure frequency reduction of 29% was associated with six sessions of progressive relaxation training (*n* = 13) compared with just 3% in patients with six sessions of quiet sitting (*n* = 11) (30).

In addition to PMR, another relaxation-based technique, yoga, has shown efficacy in seizure control for some patients with epilepsy. Yoga is an ancient Indian philosophy of life aimed at achieving the highest possible functional harmony between body and mind, which involves control of posture, breathing and meditation. Yoga may have an effect on the probability of seizure occurrence due to its effect on brain wave activity and arousal levels (39). It has been further suggested that yoga may stimulate the vagus nerve which may in turn decrease seizure frequency (40). Studies have demonstrated that certain types of yoga increase central parasympathetic activity and sensory motor rhythm and increase the sense of well-being (41, 42). In a study by Sathyaprabha and colleagues, 10 weeks of daily 60-min yoga practice (*n* = 18 epilepsy patients) was found to significantly decrease seizure frequency and parasympathetic dysfunction, compared to a group that practiced routine exercises involving sitting quietly for 20 min and simple physical exercises for 40 min who showed no reduction (*n* = 16) (32). In a further RCT by Lundgren et al. (22), drug-resistant epilepsy patients treated with either yoga (*n* = 8) or acceptance and commitment therapy (a cognitive technique; *n* = 10) experienced reduced seizure frequency and duration and increased quality of life compared to baseline (22).

Finally, training patients to relax using biofeedback has shown promise in controlling seizures. Biofeedback is a management tool where the patient is provided with visual or audio feedback to covert physiological responses occurring in their body, including changes in heart rate, skin conductivity or brain activity (from electroencephalography; EEG). Training may allow the patient to learn to actively control these bodily responses and use this biofeedback training as a countermeasure when they are aware a seizure is about to start (33). Negative amplitude shifts of cortical potential are related to seizure activity in epilepsy; regulation of the cortical potential with biofeedback has been successfully used to reduce the frequency of some patients' seizures (43). Galvanic Skin Response (GSR) is a sensitive measurement of autonomic arousal and physiological state which reflects one's behavior; a high skin resistance state reflects a state of relaxation and a low skin resistance state reflects a state of arousal. Nagai et al. found that GSR biofeedback training reduced seizure frequency in the biofeedback (*n* = 10) but not the sham control biofeedback (*n* = 8) group, thus demonstrating the promise of this technique (33). In addition, a meta-analysis of EEG biofeedback studies for epilepsy management found a 74% reduction in weekly seizures (44). This method is currently out of the scope of potential treatment techniques for canine patients, however if canine ambulatory EEG technology improves and becomes more widespread in the future, this may be another method to consider.

### Behavioral Therapy Techniques

In addition to relaxation-based interventions, a variety of behavioral therapy based techniques have been trialed in humans with epilepsy, as techniques to improve both seizure control, treat psychiatric co-morbidities and more improve QoL more generally. In a crossover design study, Gillam compared the effect of two psychological approaches: one treatment focused on seizures, and involved training patients to identify factors that might precipitate seizures, to learn how to avoid these “triggers,” to try to interrupt seizures in the early stages of their occurrence, or to practice relaxation and breathing exercises, the other targeted psychological problems affecting the patients such as phobias, mild depression, and family tensions (34). Both techniques were effective in reducing psychological symptoms and seizure frequency, with the author concluding that the improved sense of self-control may have been more important than whether the intervention was targeted at psychological difficulties or seizures specifically. In a further study, Spector et al. (35) employed a group intervention in adults with drug-resistant epilepsy (*n* = 8). The intervention, based on a programme developed by Andrews and Reiter, “*Taking Control of Your Epilepsy*” (45) and recently updated in 2015 (46) involved a variety of measures including support and dealing with stigma, understanding epilepsy and antiepileptic drugs, identifying auras, identifying and avoiding triggers for seizures, channeling negative emotions into productive outlets, and dealing with stress (35). Following treatment there was a significant reduction in seizure frequency, which persisted at 8 week follow up (35).

Behavioral medicine in the form of cognitive behavioral therapy (CBT) has further been explored as an intervention for epilepsy, with mixed results to date. CBT emphasizes the influence of thoughts and their content on emotional state and treatment focuses on changing thought patterns and behavior. In a study of *n* = 37 adults with epilepsy, patients were assigned to either a 6 week CBT program or a control group. Seizure frequency was significantly reduced in the CBT group compared to the control (36). Conversely in earlier studies, although beneficial effects upon general QoL were found, positive effects on seizure frequency were not. A recent study assigned adults with epilepsy (*n* = 17) to either a treatment group (*n* = 7) consisting of 8 weekly 2-h group CBT sessions, or to a waitlist control group (*n* = 9). Although the treatment group showed improvements in QoL [as measured by the QOLIE-31 (47)], no significant reduction in seizure frequency was found in the CBT group (37). Similar findings were reported in an earlier study (38), where no evidence to support the use of CBT for seizure reduction was found. In that study, *n* = 27 adults with epilepsy were assigned to one of three groups: CBT, supportive counseling (attention-placebo control) and waiting list (no treatment control). No difference was found in seizure frequency between the three groups; however, ratings of psychological adjustment were found to improve for the two therapy groups (38).

### Treatment of Epilepsy Co-morbidities

As highlighted earlier, epilepsy treatment should not solely focus on seizure control. Behavioral interventions may reap benefits in addition to reduced seizure frequency. In a recent Cochrane review of psychological and behavioral treatments for health-related quality of life in epilepsy patients (rather than seizure frequency as the primary outcome) (48), 24 randomized controlled trials (RCTs) were included, of which 11 were considered psychological interventions including cognitive, behavioral and mindfulness-based interventions. The impact of these interventions on QoL, as measured by a validated scale [the QOLIE-31 (47)] was significant and exceeded the threshold of minimally important change, indicating a clinically meaningful post-intervention improvement of QoL (48). Overall QoL, including mental health should be taken seriously in addition to seizure frequency in epilepsy patients, with inter-ictal anxiety and depression found to have greater adverse effects on QoL than seizure frequency, severity or chronicity in people with epilepsy (49). With anxiety found more commonly as a comorbidity in drug-resistant dogs (2), finding appropriate treatments to reduce the effects of behavioral comorbidities and improve general QoL alongside improved seizure control should be the goal of epilepsy treatment.

## Is there a Need for Behavioral Therapy in Canine Epilepsy Patients?

In addition to seizure control, there is an emerging need for therapies for co-morbid anxiety in dogs with epilepsy. Increases in anxiety following the onset of epilepsy have been documented in dogs with epilepsy (2); however, in a recent review on psychopharmacological options for the treatment of anxiety in dogs with IE, it was concluded that there is a scarcity of evidence of the effects of standard anxiolytic therapies in the IE population, with many drugs having pharmacokinetic interactions with AEDs, or contraindicated in these patients (50). Furthermore, patient response to anxiolytic medications may differ between healthy brains and epileptic brains. Although seizure reducing effects were documented in a recent study of imepitoin (an anti-consultant and anxiolytic), no changes in anxiety behavior were observed in dogs with IE (51), whereas positive effects on anxiety have been documented in otherwise healthy dogs with fear or anxiety problems (52). In addition to pharmaceutical interventions, some owners are already employing dietary therapies aimed at epilepsy and its comorbidities. In a recent study, 1 in 10 owners of epileptic dogs that were using dietary supplements reported that their aim was to improve behavioral problems associated with epilepsy (16). The use of behavioral therapy has been advocated in this population (50); however, its efficacy has yet to be ascertained with controlled trials, or indeed reported in case reports or series as yet.

## Potential Behavioral Interventions for Canine Epilepsy Patients

Although some of the aforementioned behavioral interventions for human epilepsy patients rely upon methods based upon language or higher level cognitive processes, and may not be readily adapted to canine patients, the principles of other effective techniques may be used to design potential interventions. These include:

**“Trigger” Management**: As employed in several human behavioral intervention studies, the recognition, and subsequent avoidance of seizure triggers (seizure precipitating factors) may improve seizure control in epileptic patients (34, 35). Seizure triggers have recently been documented in two studies of canine epilepsy patients, with many of these precipitants psychological (53, 54), including general stress, changes in routine, unfamiliar places or people visiting. Developing methods to reliably identify seizure triggers and to teach owners to recognize these stimuli may allow for either their avoidance, or the use of behavioral therapy to systematically desensitize dogs to these stimuli using controlled exposure (where possible) under the guidance of a clinical animal behaviorist. Although it may not be possible to avoid all seizure triggers, some reduction in seizure frequency may be achieved by manipulation of the environment (55). In addition to canine patients diagnosed with idiopathic epilepsy, dogs with reflex epilepsy (i.e., seizure activity triggered by exposure to specific locations or situations) (56) are likely to strongly benefit from this behavioral management strategy.

**Stress Reduction Therapies**: The use of behavioral therapy principles to improve the mental health of dogs, including: improved consistency of dog-owner interactions, cessation of any punishment-based training or training devices [previously shown to be used by owners of epileptic dogs (7)], and improved predictability of the dog's life with enhanced feelings of control, may be a potential way to reduce overall stress and improve seizure control (57–59). Indeed, a previous human behavioral intervention study found no difference in seizure control between treatment programmes focused on general psychological problems *vs*. seizures themselves specifically, with the author concluding that the improved sense of self-control induced by either programme may have been more important than the specific type of intervention. Development of general stress reduction programmes for epilepsy patients has the potential to improve seizure control while also improving overall QoL of these patients by improving mental health.

**Specific Relaxation Based Activities**: There is growing evidence that dogs with epilepsy exhibit behavioral changes preceding a seizure event, which may represent the “prodromal” stage of the seizure cycle. In a study of *n* = 229 owners of dogs with epilepsy, 65% reported that dogs showed pre-seizure changes including included restlessness, clinginess and fearfulness (54). As implemented in human studies [e.g., Dahl et al. (29)], it is possible that applying acute relaxation based interventions during this pre-seizure stage may be effective in aborting impending seizure activity, if seizure generation is influenced by stress levels and cortical excitability can be dampened by relaxation based activities. At present, further research is needed to confirm the presence of this phase of the “seizure cycle” in dogs and whether this window of time for intervention exists. Relaxation sessions, aimed at reducing physiological arousal and thereby increasing the threshold for response to sensory signals, are used as a foundation for modifying context-specific behavior in many clinical behavior cases (60, 61) and daily relaxation outside of the prodromal phase may act as an anxiolytic if dogs are trained to effectively settle in a low arousal, positively valenced state.

## Conclusions

Studies of behavioral interventions as tools to improve seizure control and QoL show great promise for human drug-resistant epilepsy patients. Despite the limitations of some of the early studies in this area, with small sample sizes commonplace, the fact that significant reductions in seizure frequency were found in such small samples provides compelling preliminary evidence for the strength of these interventions.

With great similarities exhibited between drug resistant human and canine epilepsy patients (62), trialing behavioral methods for this challenging population is timely and urgently needed. There are both challenges and opportunities in the use of behavioral methods, with important lessons to be learned from the past 40 years of human research in this field. Well-designed and powered studies are needed to successfully address whether behavioral treatments can increase seizure threshold and improve seizure control in drug resistant epilepsy patients. In both veterinary and human medicine there is a need for multi-center, double-blind, placebo-controlled trials to confirm the effects of behavioral interventions on seizure frequency.

In the absence of such evidence to date, the use of established behavioral medicine techniques to reduce stress and improve mental health of these often sensitive and challenging patients is advocated. Basic principles for all patients include the cessation of all punishment-based training techniques employed by owners. Greater education for owners on both overt and subtle signs of stress in their dog is essential for all behavioral interventions, and vets should either advise clients individually of these signs, or be able to direct owners to appropriate, evidence-based resources on this topic. We advocate a greater role for veterinary behaviorists in the management of epilepsy patients alongside neurologists and general practitioners, not only for epileptic patients with overt behavioral problems that owners actively seek treatment for (e.g., noise phobias), but also for expert opinion on a dog's behavioral husbandry and lifestyle with the aim of generally improving of the mental health and QoL of these sensitive patients.

## Author Contributions

The idea for the manuscript was devised by RP. RP, SH, and EB all contributed ideas to and reviewed the final manuscript.

### Conflict of Interest Statement

The authors declare that the research was conducted in the absence of any commercial or financial relationships that could be construed as a potential conflict of interest.
